# In vitro effects of interleukin (IL)-1 beta inhibition on the epithelial-to-mesenchymal transition (EMT) of renal tubular and hepatic stellate cells

**DOI:** 10.1186/s12967-019-1770-1

**Published:** 2019-01-07

**Authors:** Valentina Masola, Amedeo Carraro, Simona Granata, Lorenzo Signorini, Gloria Bellin, Paola Violi, Antonio Lupo, Umberto Tedeschi, Maurizio Onisto, Giovanni Gambaro, Gianluigi Zaza

**Affiliations:** 1 Renal Unit, Department of Medicine, University-Hospital of Verona, Piazzale A. Stefani 1, 37126 Verona, VR Italy; 2 Department of General Surgery and Odontoiatrics, Liver Transplant Unit, University Hospital of Verona, Piazzale Stefani 1, 37126 Verona, Italy; 3 Department of Biomedical Sciences, University of Padova, Via Ugo Bassi, 58/B, 35131 Padua, Italy; 4 Division of Nephrology and Dialysis, School of Medicine, Columbus-Gemelli Hospital Catholic University, Largo Agostino Gemelli 8, 00168 Rome, RM Italy

**Keywords:** Epithelial to mesenchymal transition, Canakinumab, Fibrosis, Liver, Kidney, Interleukin 1 beta

## Abstract

**Background:**

The epithelial to mesenchymal transition (EMT) is a multi-factorial biological mechanism involved in renal and hepatic fibrosis and the IL-1 beta has been assumed as a mediator of this process although data are not exhaustive. Therefore, the aim of our study was to evaluate the role of this cytokine in the EMT of renal proximal tubular epithelial cells (HK-2) and stellate cells (LX-2) and the protective/anti-fibrotic effect of its inhibition by Canakinumab (a specific human monoclonal antibody targeted against IL-1beta).

**Methods:**

Both cell types were treated with IL-1 beta (10 ng/ml) for 6 and 24 h with and without Canakinumab (5 μg/ml). As control we used TGF-beta (10 ng/ml). Expression of EMT markers (vimentin, alpha-SMA, fibronectin) were evaluated through western blotting and immunofluorescence. Genes expression for matrix metalloproteinases (MMP)-2 was measured by Real-Time PCR and enzymatic activity by zymography. Cellular motility was assessed by scratch assay.

**Results:**

IL-1 beta induced a significant up-regulation of EMT markers in both cell types and increased the MMP-2 protein expression and enzymatic activity, similarly to TGF-beta. Moreover, IL-1 beta induced a higher rate of motility in HK-2. Canakinumab prevented all these modifications in both cell types.

**Conclusions:**

Our results clearly demonstrate the role of IL-1 beta in the EMT of renal/stellate cells and it underlines, for the first time, the therapeutic potential of its specific inhibition on the prevention/minimization of organ fibrosis.

**Electronic supplementary material:**

The online version of this article (10.1186/s12967-019-1770-1) contains supplementary material, which is available to authorized users.

## Background

Renal and hepatic fibrosis are characterized by the accumulation of extra-cellular matrix (ECM), and both represent the final common pathway of a wide variety of renal and liver diseases. In this context, epithelial–mesenchymal transition (EMT) has a central role.

During EMT epithelial cells lose their junctions, the apical–basal polarity and their epithelial regular shape acquiring high motility, the ability to produce ECM, and apoptosis resistance [1–3]. During the trans-differentiation, these cells undergo a reprogramming of gene expression with down-regulation of epithelial markers, up-regulation of mesenchymal markers (Vimentin, α-SMA) and ECM components (type I collagen and Fibronectin) [4].

This process is regulated by a complex intracellular network, where specific transcription factors (SNAIL, SLUG, ZEB1), surface molecules, cytoskeleton proteins and matrix metalloproteinases (MMP-2, MMP-9) are involved, as well as several miRNAs [1]. Numerous biological elements are able to induce EMT, such as growth factors (e.g., epidermal growth factor, fibroblast growth factor, connective tissue growth factor, platelet-derived growth factor, insulin-like growth factor), integrins, transforming growth factor β (TGF-β) e cytokines (e.g., TNF-α, IL-4, IL-13) [5, 6].

However, in the biological mechanisms underlie both renal and liver fibrosis, IL-1β, a pro-inflammatory mediator derived mainly by macrophages, endothelial cells, epithelial cells and fibroblasts seems to have a central role. Major effects of the IL-1 beta include: (1) Endothelial cells activation; (2) Neutrophils diapedesis induction; (3) Enhancement of lymphocytes (T and B) cytokines synthesis [7].

Under physiological conditions, tissues do not express IL-1β but it can be rapidly induced through the activation of pattern recognition receptors (e.g. toll-like receptors, TLR) activated by pathogens and damaged cells products [8].

IL-1β is first synthesized as biologically inactive pro-IL-1β and then processed into biologically active IL-1β through a caspase-1-dependent proteolysis [9, 10]. Since CASPASE-1 is usually inactive, an effective IL-1β secretion is finely regulated and depends on inflammasome activation, a complex constituted by NOD-like receptors, ASC and the CASPASE-1 itself [11]. Moreover, IL-1β can be activated through mast cell-derived proteases and neutrophils-derived elastase and cathepsin-G [12, 13].

This cytokine exerts some pro-fibrotic effects by inducing TGF-β synthesis [14, 15] pro-inflammatory cytokines and fibrosis markers (i.e. FIBRONECTIN, α-SMA) release [15], and activating fibroblasts proliferation [16, 17]. Interestingly, fibroblasts seem to increase their sensitivity to this interleukin over the time, as shown in cells from fibrotic kidneys [16, 18].

Moreover, IL-1β expression can be correlated with the degree of glomerulosclerosis [19–24] and, as demonstrated in several animal models, its inhibition can slow down the progression of chronic renal damage. Furthermore, the administration of an IL-1R antagonist completely abrogated interstitial fibrosis/tubular atrophy (IF/TA) [25–27]. In a murine model of unilateral ureteral obstruction, IL-1β has demonstrated itself to be essential for synthesis and release of TGF-β and its downstream effects including the expression of connective tissue growth factor (CTGF) and type I COLLAGEN synthesis [28].

Also in hepatic tissue, IL-1β is an important and early mediator of fibrosis. It is induced within 1 h after the pro-fibrotic insult, and, stimulating MMP-9 expression, mediates the ECM degradation within Disse’s space leading to collapse of hepatic sinusoids [29–32]. In addition, IL-1β promotes survival of activated hepatic stellate cells (HSCs) [33]. As a consequence, IL-1R antagonism showed itself to have a protective anti-fibrotic role also in hepatic tissues [31, 34].

On these bases, several IL-1 targeting agents (such as Canakinumab, Anakinra and Rilonacept) have been developed and introduced in clinical practice that interfere with the bond between IL-1β, its receptor (IL-1R), and the accessory protein of this receptor (IL-1RacP) [35].

Among them, Canakinumab (ACZ885, Ilaris^®^, Novartis), a human monoclonal anti-body (mAb) targeting IL-1β, may represent a new pharmacological tool to treat and minimize complications following several chronic degenerative diseases [36–39].

Canakinumab presents some interesting characteristics, which make it profitable with respect to other IL-1β inhibitors: it is highly specific for IL-1β, whereas Anakinra and Rilonacept act against both IL-1α and β. Furthermore, it has a long half-life, which allows low-dose and long-lasting administration [40].

Therefore, the aim of this study has been to evaluate the effect of Canakinumab on IL-1β-induced EMT in kidney epithelial and hepatic stellate cells. This could reinforce the available literature regarding its potential use in clinical practice.

## Materials and methods

### Cellular cultures and treatments

Renal proximal tubular epithelial cells (HK-2) was purchased from American Type Culture Collection (ATCC) and human stellate hepatic cells (LX-2) was obtained from Merck Millipore (Germany). Both cell lines were cultured in DMEM-High Glucose (EuroClone) (17.5 mM glucose) with 10% fetal bovine serum (Biochrom AG), l-glutamine (2 mM), penicillin (100 U/ml) and streptomycin (100 μg/ml) at 37 °C in a humidified atmosphere with 5% CO_2_. After reaching a confluence of about 80%, serum was removed for 24 h (starvation) and subsequently treated with TGF-β (10 ng/ml) or IL-1β (10 ng/ml) [41, 42] in the presence or absence of Canakinumab (5 μg/ml).

### Migration assay

HK-2 cells monolayer was incised with a sterile pipette tip to create a scratch and washed with PBS to remove non-adherent cells. Then it is incubated with culture media containing TGF-β, IL-1β in presence or absence of Canakinumab. The cells were photographed at different time points and the scratch area was measured in each photo to obtain a mean value. The migration was reported as the difference (in mm^2^) between the scratch area dimensions observed at baseline and after 24 h. Each experimental condition was tested in triplicate.

### Gene expression analysis

Both cell types were treated with TGF-β (10 ng/ml) or IL-1β (10 ng/ml) in the presence or absence of Canakinumab (5 μg/ml) for 6 h. Then, total RNA was extracted with Trizol reagent (Invitrogen), following the manufacturer’s instructions. Quantity and quality of RNA were checked using the Nanodrop spectrophotometer (EuroClone). Total RNA was reverse-transcribed into cDNA using the reverse transcriptase SuperScript II (Invitrogen). Real Time-PCR reactions were performed with the ABI-Prism 7500 using Power SYBR Green Master Mix 2 (Applied Biosystem) and specific primers for *Mmp*-*2* and *Glyceraldehyde*-*3*-*phosphate dehydrogenase* (*Gapdh*). The primers are listed in Table 1.

**Table 1:** Sequences of primers used for Real-Time PCR

	Forward 5′-3′	Reverse 5′-3′
*Gapdh*	ACACCCACTCCTCCACCTTT	TCCACCACCCTGTTGCTGTA
*Mmp*-*2*	GCGGCGGTCACAGCTACTT	CACGCTCTTCAGACTTTGGTTCT

The comparative Ct method (ΔΔCt) was used to quantify gene expression, and the relative quantification was calculated as 2^−ΔΔCt^. The GAPDH gene amplification was used as a reference standard to normalize the target signal. Melting curve analysis was used to confirm the specificity of amplification.

### Zymography

In order to evaluate the activity of MMP-2 in the conditioned media of HK-2 and LX-2 cells we used a zymography on a gelatin substrate. Conditioned media were prepared by incubating subconfluent cells in serum-free medium with TGF-β (10 ng/ml) or IL-1β (10 ng/ml) in the presence or absence of Canakinumab (5 μg/ml) for 24 h. Equal amounts of conditioned media were processed in SDS-PAGE under non-reducing conditions on 10% SDS-polyacrylamide gels co-polymerized with 0.5% gelatin, which is a substrate for gelatinases. After electrophoresis, gels were washed twice for 30 min in 2.5% Triton X-100 at room temperature to remove SDS, then equilibrated for 30 min in collagenase buffer. Finally, these gels were incubated overnight with a new collagenase buffer at 37 °C. After incubation, the gels were stained in 0.1% Coomassie Brilliant Blue R-250, 30% MetOH/10% acetic acid for 1 h and destained in 30% MetOH/10% acetic acid. The digestion bands were analyzed using ImageJ software.

### Western blotting

After 24 h of treatment, cells were lysed in a buffer (50 mM Tris–HCl pH 5.5, 150 mM NaCl, Triton X-100 0.5%) added with a complete protease inhibitor (Roche Applied Science). Equal amounts of proteins were denatured at 100 °C for 10 min and subjected to SDS-PAGE on 10% polyacrylamide gel. After the run, the proteins were electro-transferred on nitrocellulose membranes. Saturation of non-specific sites was performed for 2 h at room temperature in 5% milk in the TBST buffer (50 mM Tris–HCl, pH 7.4, 150 mM NaCl, 0.1% Tween 20). The membranes were exposed to primary antibodies directed against GAPDH (Santa Cruz sc-25778), α-SMA (Sigma A5228), VIMENTIN (VIM) (Santa Cruz 7557), FIBRONECTIN (FN) (Santa Cruz sc-9068) overnight at 4 °C and subsequently incubated with a secondary antibody conjugated with peroxidase for 1 h at room temperature. The signal was detected with Luminata™ Forte Western HRP Substrate (Millipore) according to the manufacturer’s instructions and the signal was acquired with Mini HD9 (UVItec, Cambridge). The band intensities were quantified using the UVItec Image Program.

### Immunofluorescence

Both cell types, treated as described above, were seeded in CultureSlides (Falcon) to analyze the expression of α-SMA, VIM proteins and stress fibers. These cells, fixed in 4% paraformaldehyde and permeabilized, were incubated overnight at 4 °C in PBS with 1% bovine serum albumin (BSA) with the primary antibodies directed against α-SMA (1A4, Sigma) and VIM (Santa Cruz 7557). Then, the cells were washed with PBS prior to incubation for 1 h at room temperature with secondary antibodies (Goat anti-mouse IgG Alexa Fluor 546, Donkey anti-goat IgG Alexa Fluor 488) in 1% PBS/BSA. Cytoskeleton was visualized with phalloidin-TRITC (P1951, Sigma-Aldrich). The nuclei were marked with Hoechst 33258. A LeicaSP5 confocal microscope acquired the images. Exposure times and illumination intensity were the same for all the images.

### Oil Red O staining

Cells were seeded in CultureSlides (Falcon), cultured to sub-confluence treated as described previously and fixed in 4% paraformaldehyde. Fixed cells were incubated with Oil Red O working solution for 1 h, then cells were washed in PBS. Nuclei were counterstained with eosin.

### Statistical analysis

Mean ± S.D. of the real-time PCR data were calculated with Rest2009 software. Differences between control (CTR) and TGF-β or IL-1β treated cells or between pre- and post-treatment with Canakinumab, were compared using two-tailed Student’s t-test by R software (version 3.5.1). A p-value < 0.05 was set as the level of significance for all tests.

## Results

### Effects of IL-1β inhibition on EMT of renal proximal tubular epithelial cells (HK-2)

To evaluate the potential anti-fibrotic effect of the IL-1β inhibitor, Canakinumab (CANAK), we measured by western blotting the protein level of three well-known EMT markers in HK-2 cells exposed to TGF-β or IL-1β in presence or absence of this agent.

**Fig. 1:**
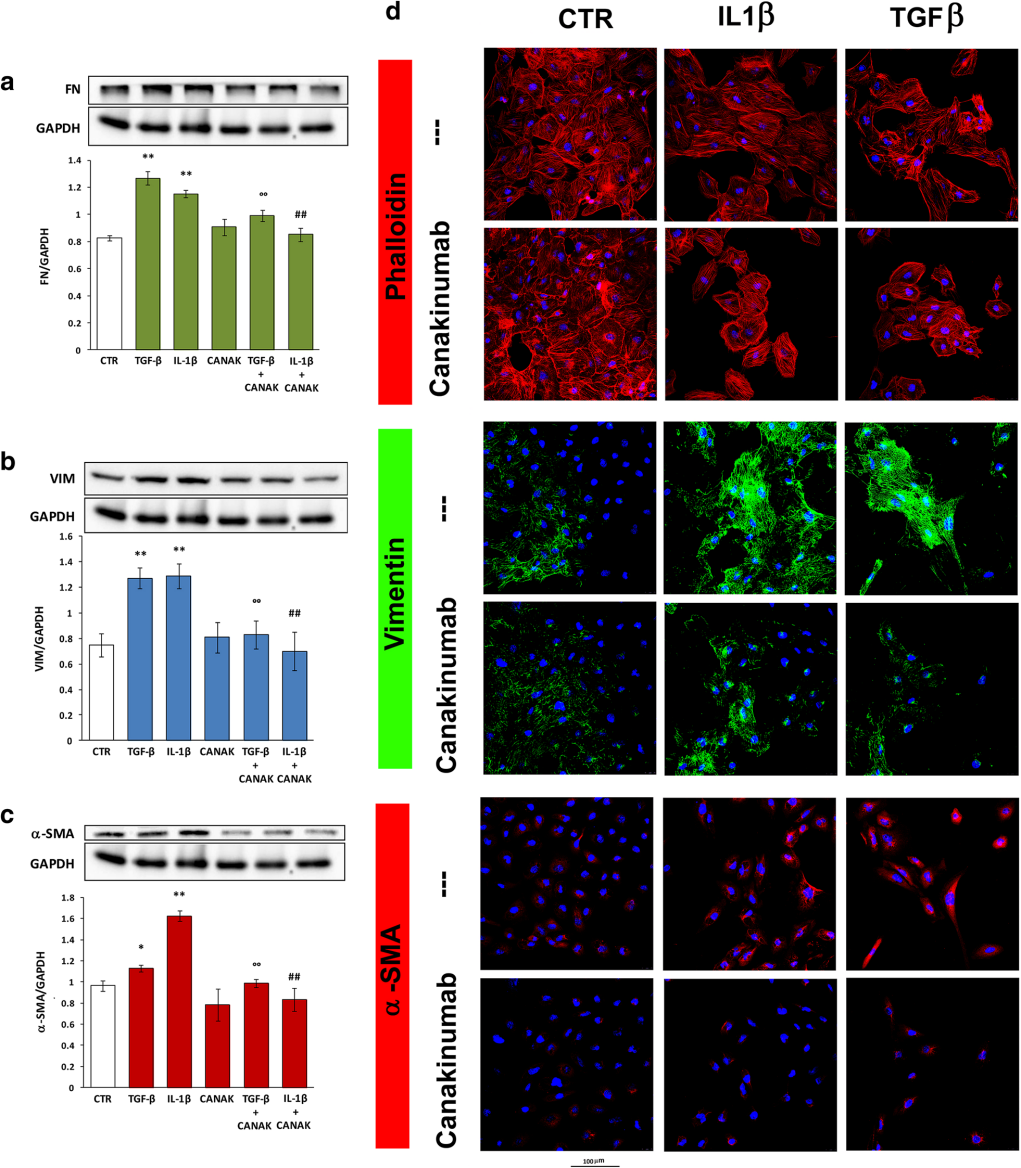
Protein level of EMT markers in HK-2 cells treated with TGF-β, IL-1β and Canakinumab. Protein expression of **a** FN, **b** VIM and **c** α-SMA in HK-2 cells treated with TGF-β (10 ng/ml) or IL-1β (10 ng/ml) with and without Canakinumab (5 μg/ml). Upper part: representative western blotting of all the experiments. Lower part: histograms showing mean ± SD of protein expression levels normalized to GAPDH of three experiments. *p < 0.05, **p < 0.01 vs control cells (CTR); ^°°^p < 0.01 vs TGF-β; ^##^p < 0.01 vs IL-1β. **d** Representative immunofluorescence images from three independent experiments for phalloidin, VIM and α-SMA in HK-2 cells treated with IL-1β or TGF-β in presence and absence of Canakinumab (Zoom × 400)

In HK-2 cells, IL-1β caused a significant increase in FN, VIM, and α-SMA levels, comparable to those induced by TGF-β (used as a control) (Fig. 1a–c). The addition of the CANAK prevent this effect. Interestingly, its protective effect also occurred in the presence of TGF-β.

Immunofluorescence experiments performed on HK-2 under the same conditions confirmed that IL-1β and TGF-β increased the protein expression of VIM and α-SMA. Phalloidin staining revealed that IL-1β and TGF-β reduced the pool of junctional actin fibers and increased the stress fiber formation (Fig. 1d). The addition of Canakinumab inhibited the above-described effects.

### HK-2 cells migration in presence of IL-1β

**Fig. 2:**
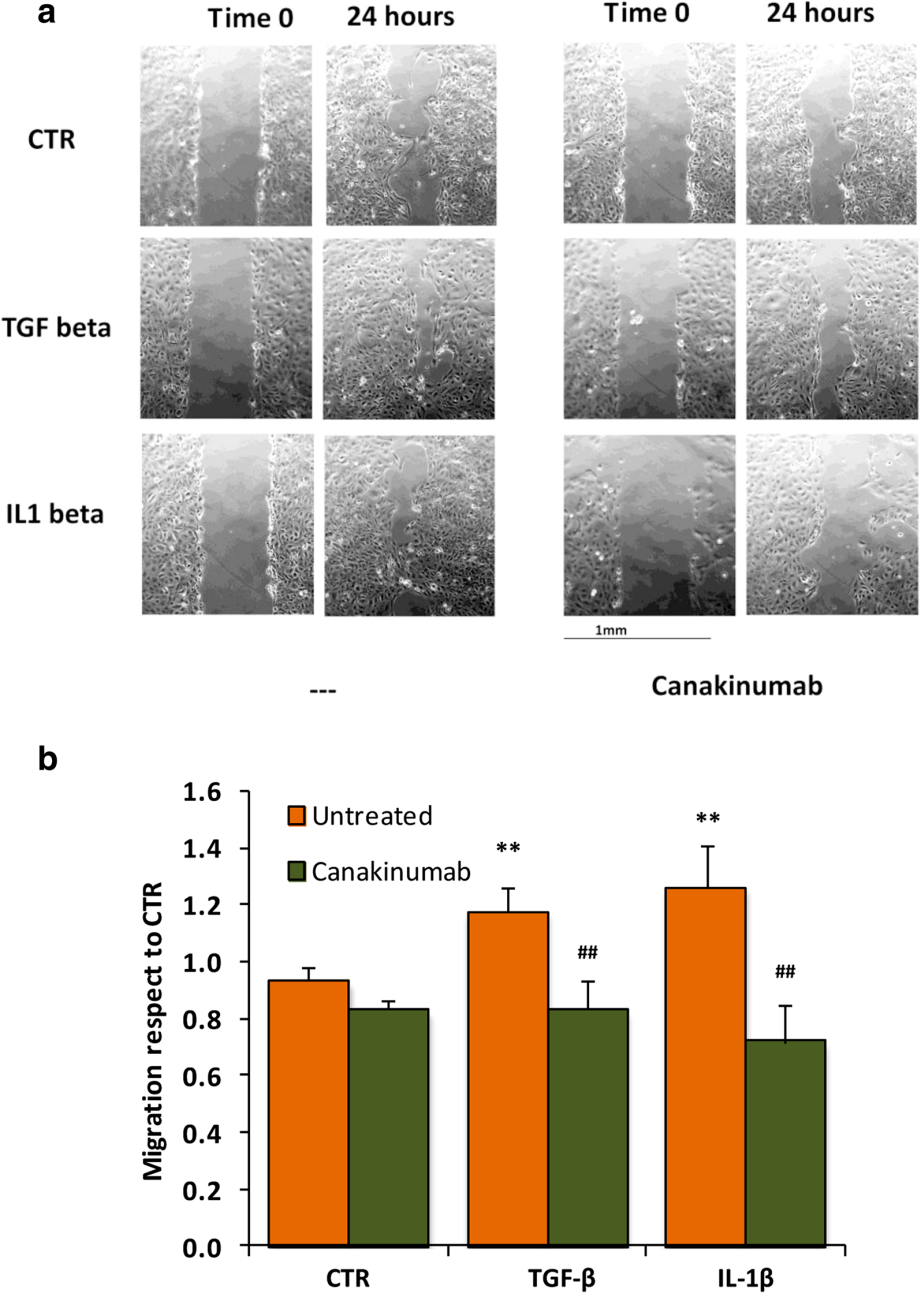
Canakinumab reduces the cellular motility of HK-2 cells treated with IL-1β. **a** Image representative of cells at time 0 and after 24 h of treatment with IL-1β or TGF-β with and without Canakinumab. **b** Histogram represents migration (in mm^2^) over 24 h of HK-2 cells before and after treatment with IL-1β or TGF-β in presence and absence of Canakinumab. Data are indicated as mean ± SD of three experiments. *p < 0.05, **p < 0.01 vs untreated control cells (CTR), ^##^p < 0.01 vs untreated

To confirm the ability of renal tubular epithelial cells to migrate through the basal membrane into the interstitium (prerogative effect of EMT), we performed a cell motility assay. As expected, we demonstrated that IL-1β (similarly to TGF-β, used as a control) significantly increased the motility of HK2 cells (Fig. 2) confirming a possible differentiation toward a mesenchymal phenotype. Canakinumab inhibited this effect also when mediated by TGF-β.

### Metalloproteinase-2 regulation in HK-2 cells

**Fig. 3:**
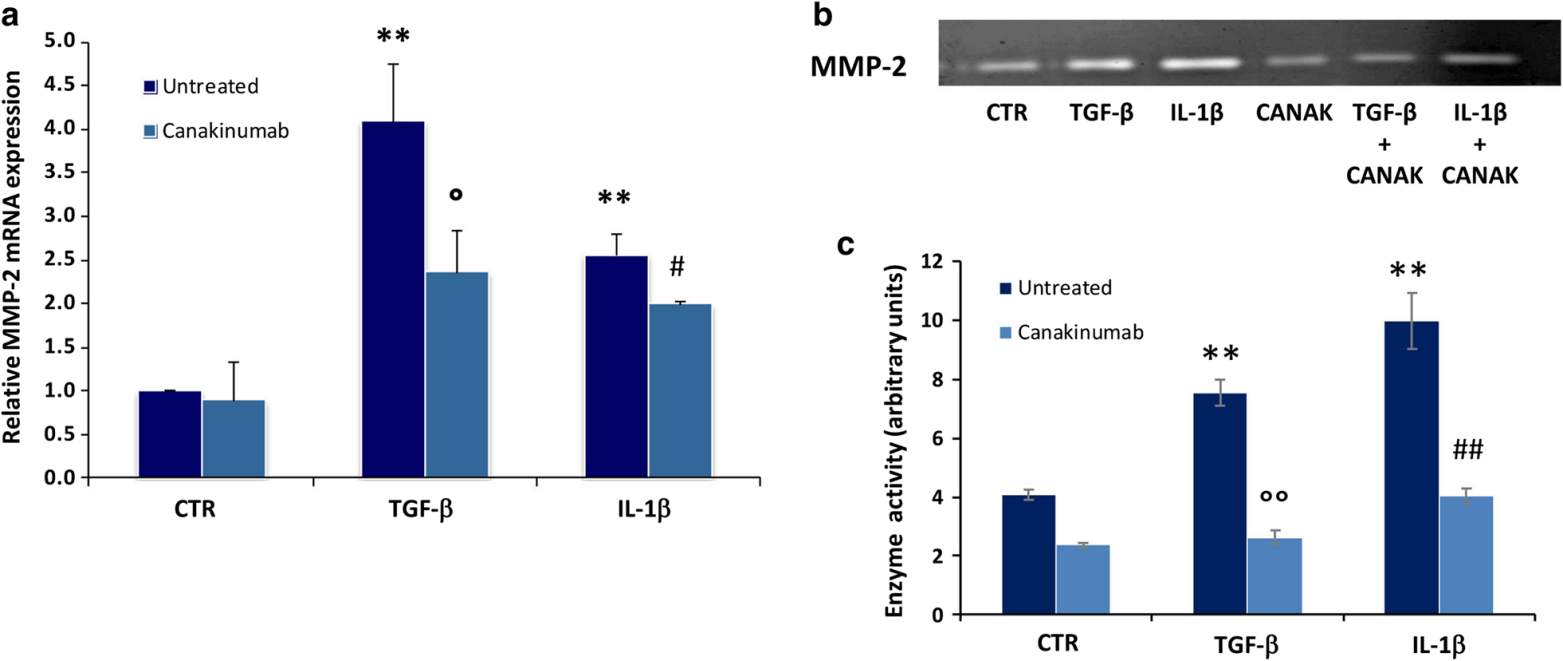
Canakinumab reduces Mmp-2 expression and enzymatic activity induced by IL-1β in HK-2 cells. **a** Gene expression of *Mmp*-*2* measured by Real Time-PCR in HK-2 cells treated with IL-1β or TGF-β with and without Canakinumab. Expression levels are normalized to *Gapdh*. Data are indicated as mean ± SD of three experiments performed in triplicate. **b** Gelatin zymography shows the activity of MMP-2 in the conditioned media of HK-2 cells treated for 24 h with TGF-β (10 ng/ml) or IL-1β (10 ng/ml) in the presence or absence of Canakinumab (5 μg/ml). **c** The histogram represents the densitometric analysis of enzymatic activity of MMP-2 as a mean ± SD of three experiments. The p value was calculated with the t-test. **p < 0.001 versus untreated control cells (CTR); ^°^p < 0.05, ^°°^p < 0.01 vs TGF-β untreated; ^#^p < 0.05, ^##^p < 0.01 vs IL-1β untreated

To clarify the role of IL-1β as inducer of EMT and to underline the anti-fibrotic effect of Canakinumab, we measured by Real-Time PCR the gene expression of a well-known EMT mediator responsible for extracellular matrix accumulation during fibrosis: *Mmp*-*2*. The results of our experiments clearly showed that IL-1β increases the gene expression of *Mmp*-*2* (Fig. 3a). Interestingly, we found that this cytokine has no effect on *Mmp*-*9* gene transcription levels (Additional file 1: Figure S1), according to previous studies [43, 44].

To confirm the inhibitory effects of IL-1β on MMP-2, we evaluated its enzymatic activity by zymography. Results showed that IL-1β (similarly to TGF-β) induced a significant increase in the MMP-2 enzymatic activity of this metalloproteinase. Contrarily, the presence of the Canakinumab prevented this effect (Fig. 3b, c). Although IL-1β has no effect on Mmp-9 gene expression, the zymography revealed an increment in its activity hampered by Canakinumab (Additional file 1: Figure S2).

### Effect of the IL-1β inhibition on the expression of EMT markers in hepatic stellate cells (LX-2)

To validate the results obtained in renal cells and to clarify the pro-fibrotic role of this cytokine in liver, a highly prevalent condition with a great clinical impact, we performed in vitro experiments by employing hepatic stellate cells (LX-2).

Similarly to HK-2, we evaluated the impact of IL-1β and TGF-β to up-regulate EMT markers (α-SMA, VIM and FN) and the inhibitory potential of Canakinumab.

**Fig. 4:**
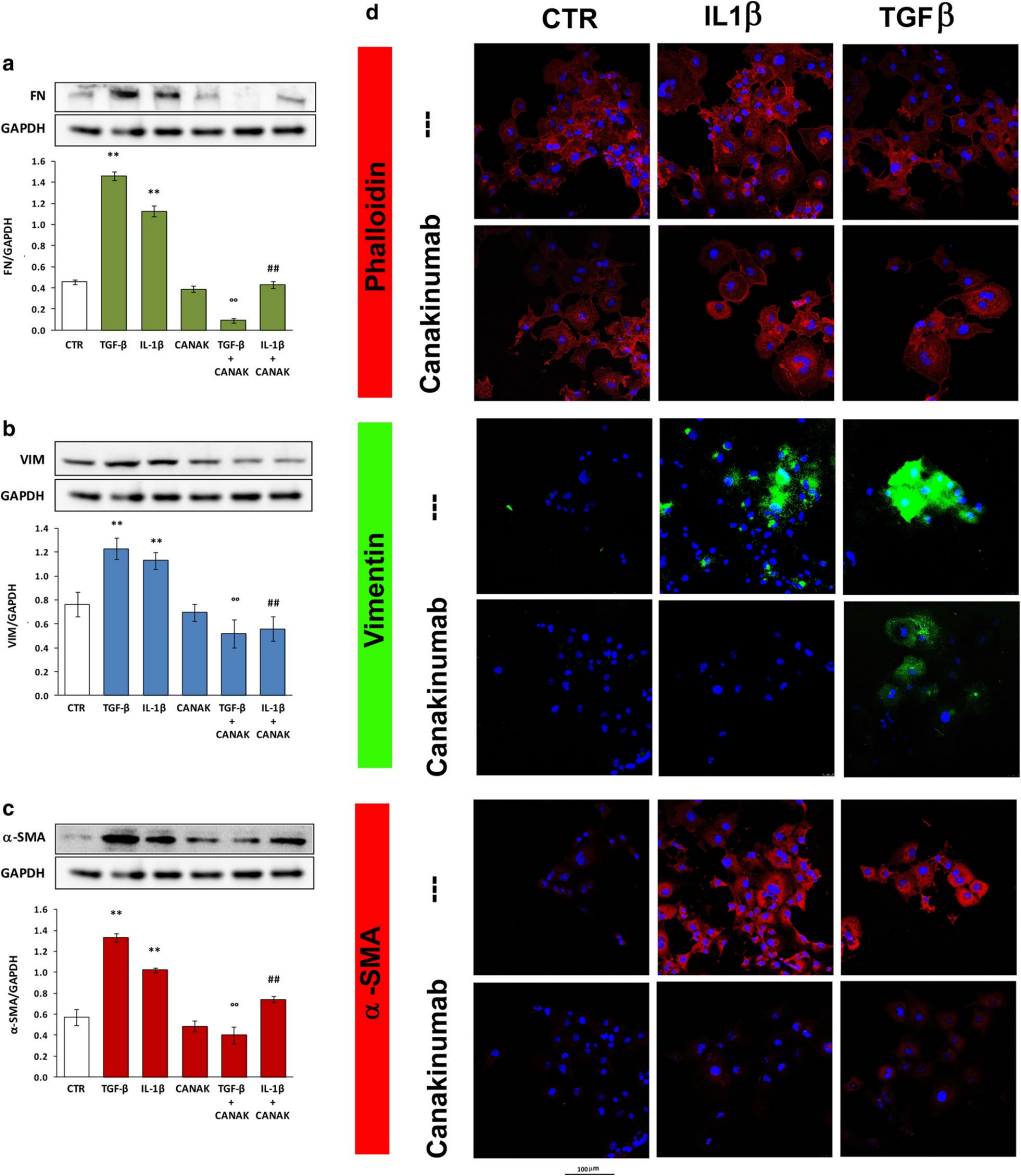
Protein level of EMT markers in LX-2 treated with TGF-β, IL-1β and Canakinumab. Protein expression of **a** FN, **b** VIM and **c** α-SMA in LX-2 treated with TGF-β (10 ng/ml), IL-1β (10 ng/ml) with and without Canakinumab (5 μg/ml). Upper part: representative western blotting of all the experiments. Lower part: histograms showing mean ± SD of protein expression levels normalized to GAPDH. **p < 0.01 vs control cells (CTR); ^°°^p < 0.01 vs TGF-β; ^##^p < 0.01 vs IL-1β. **d** Representative immunofluorescence images from three independent experiments for phalloidin, VIM and α-SMA in LX-2 cells treated with IL-1β or TGF-β in presence and absence of Canakinumab (Zoom × 400)

Western blot analysis revealed that IL-1β was able to induce (similar to TGF-β) an over-expression of all the above-mentioned markers and the presence of Canakinumab inhibited these pro-fibrotic effects (Fig. 4a–c). As a further confirmation of these results, we performed immunofluorescence experiments for Phalloidin, VIM and α-SMA in cells treated with IL-1β and Canakinumab obtaining similar results (Fig. 4d).

### Metalloproteinases expression/activity in LX-2 cells

**Fig. 5:**
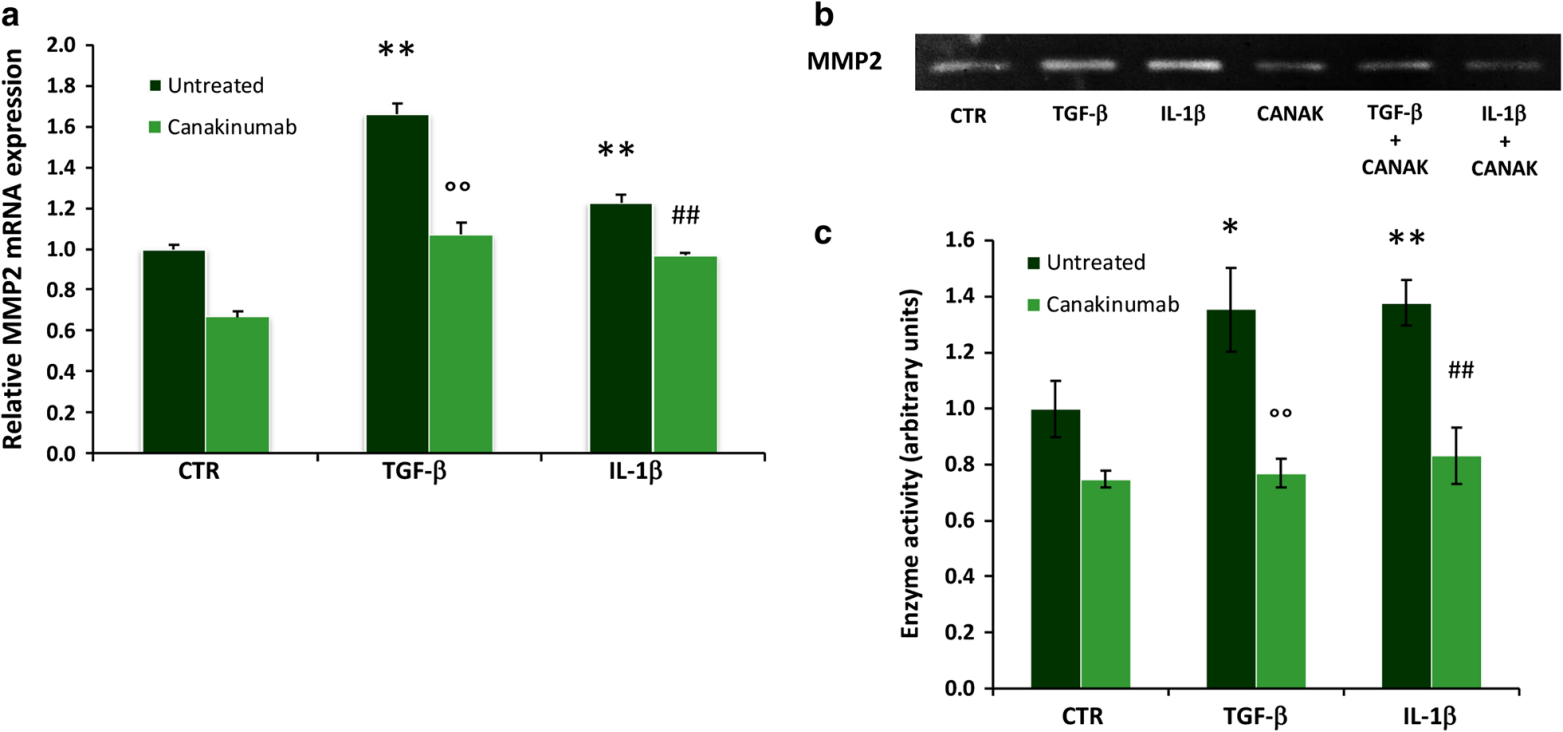
Canakinumab reduces MMP-2 expression and enzymatic activity induced by IL-1β in LX-2 cells. **a** Gene expression of *Mmp*-*2* evaluated by Real-Time PCR in LX-2 cells treated with TGF-β (10 ng/ml), IL-1β (10 ng/ml) in presence and absence of Canakinumab. Expression levels were normalized to *Gapdh*. Mean ± SD of three different experiments performed in triplicate. **b** Gelatin zymography shows the activity of MMP-2 in the conditioned media of HK-2 cells treated for 24 h with TGF-β (10 ng/ml) or IL-1β (10 ng/ml) in the presence or absence of Canakinumab (5 μg/ml). **c** Histogram represents the densitometry analysis of the enzymatic activity as a mean ± SD of three experiments performed in triplicate. The p value was calculated with the t-test. *p < 0.05, **p < 0.01 versus untreated control cells (CTR); ^°°^p < 0.01 vs TGF-β untreated; ^##^p < 0.01 vs IL-1β untreated

Similarly to HK2 cells, in LX-2 the administration of IL-1β was able to increase metalloproteinase 2 (Fig. 5a) at gene expression level. Instead, Canakinumab maintained mRNA levels of this metalloprotease at levels comparable to controls (Fig. 5a). Likewise mRNA level of Mmp-9 was augmented by IL-1β and Canakinumab prevent this effect (Additional file 1: Figure S3).

Additionally, similarly to HK-2, the enzymatic activity of MMP-2 after IL-1β and TGF-β stimulation was increased compared to controls. Canakinumab treatment inhibited this effect (Fig. 5b, c). MMP-9 activity was not detectable in this cell line (Additional file 1: Figure S4).

### Activation of LX-2 cells after Canakinumab

**Fig. 6:**
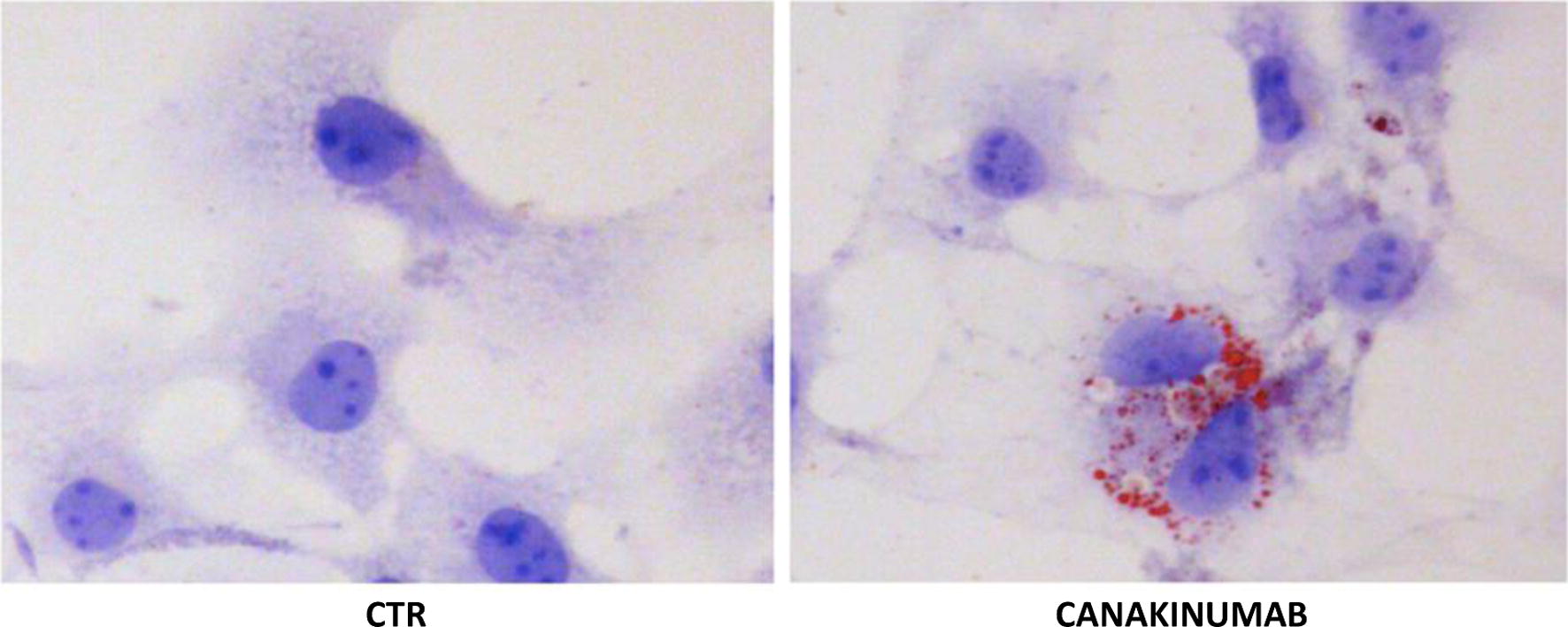
Inactivation of HSCs by the Canakinumab. Staining with Oil Red O shows an increase in the content of lipid droplets in LX-2 cells treated with Canakinumab. The inhibition of IL-1β by Canakinumab promotes the inactivation of HSCs as demonstrated by the accumulation of lipid droplets in the cytoplasm. The images are representative of one of three experiments performed with Oil Red O staining

Since the number and size of lipid droplets are inversely correlated with the activation status of LX-2 (31), we have analyzed the role of Canakinumab in reducing the lipid droplets content of LX-2. Staining with Oil Red O revealed an increase in the number of lipid droplets after treatment with Canakinumab (Fig. 6). This phenomenon suggests that the inhibition of IL-1β leads LX-2 cells into a quiescent state.

## Discussion

In the last few years, many authors have studied the mechanisms underlying the trans-differentiation of epithelial cells to a mesenchymal phenotype and searched for therapeutic agents able to minimize this process. In fact, it is known that the progression of chronic organ damage and, in particular, the development of chronic renal and hepatic disease represent an important public health problem.

In literature, it is widely described that in both kidney and liver, EMT is an active process that occurs through the loss of the epithelial phenotype of many cellular subsets with the over-expression of specific mesenchymal surface markers, such as vimentin and α-smooth muscle actin [3].

In addition, because of the cytoskeletal remodelling and release of matrix metalloproteases with consequent basal membrane degradation, cells with acquired myofibroblastic phenotype are able to migrate into the interstitium, where they can play a key role in the pathogenic process leading to chronic renal damage [1]. These processes could also represent important determinants of the metastatic capacity of tumor cells.

Among the biological/biochemical elements inducing EMT there are several growth factors, integrins and cytokines such as TNF-α, IL-4, IL-13 and IL-1β. In particular, the role of IL-1β and its receptor (IL-1R) targeting agents in renal and hepatic fibrosis has been evaluated in a few animal model studies. In particular, in models of glomerulonephritis and unilateral ureteral obstruction, the administration of a specific IL-1R antagonist was able to inhibit the progression of functional damage and almost completely abrogated interstitial fibrosis/tubular atrophy (IF/TA) [25–27].

Similarly, in mouse models of liver injury, IL-1R antagonist or silencing caused a reduction in laminin and collagen deposition in the tissue and a reduction of inflammatory infiltrate compared to untreated or wild type animals [31, 34].

Therefore, to confirm the role of IL-1β in the kidney and liver and to analyze, for the first time, the potential protective effect of Canakinumab, a monoclonal antibody targeting IL-1β currently used for the treatment of immune-inflammatory diseases (such as rheumatoid arthritis, systemic juvenile idiopathic arthritis, and syndromes associated with cryopyrin), we treated epithelial cells of the proximal renal tubule (HK-2) and hepatic stellate cells (LX-2) with this cytokine and subsequently measured EMT markers using various classical molecular biology techniques.

As expected, in both cell lines the IL-1β stimulus determined a specific phenotypic change towards a mesenchymal type. Furthermore, inhibition with Canakinumab minimized this pro-fibrotic effect.

Our results, on the other hand, clearly showed for the first time the anti-fibrotic role of Canakinumab occurred on cells treated with TGF-β, a well-known inducer of EMT and fibrosis. This finding was in agreement with some literature data that reported a synergistic effect of IL-1β and TGF-β in collagen deposition as well as in the FN accumulation in mesangial cells [45, 46].

Moreover, we found an interesting effect of IL-1β on the two most important matrix metalloproteases (MMP-2 and MMP-9) involved in organ fibrosis. In both cell lines, IL-1β increased expression and enzymatic activity of MMP-2. Interestingly in renal cells, although IL-1β had no effect on Mmp-9 gene expression, the zymography revealed an increment in its activity hampered by Canakinumab probably via a post-transcriptional modification mechanism. In contrast, in liver cells Canakinumab inhibited the up-regulation of Mmp-9 mRNA level but its enzymatic activity was not detectable. Although other authors have already observed this effect, the mechanism is not yet fully understood. It seems to involve the activation of several mitogen-activated protein kinases (MAPK). This is a family of serine/threonine kinases mediating cellular response to extracellular stimuli, such as stress, oncogenes, mitogens and inflammation, through regulation of gene expression, mitosis, metabolism, survival, proliferation and apoptosis [43, 44, 47]. Further studies are needed to confirm these data and to clarify the mechanisms behind this different response.

Overall, our results illustrate new therapeutic applicability of antibodies targeted against IL-1β, which is added to the well-described anti-inflammatory potential. In addition, they could prove to be good therapeutic weapons for chronic pro-fibrotic diseases.

In fact, Niccoli et al. have recently reported a beneficial effect of Canakinumab on a patient suffering from asbestosis, a progressive interstitial lung disease caused by inhalation of asbestos fibers that occurs in subjects long-exposed to asbestos dust (miners, quarry workers, millers) [48]. It is plausible that this positive result was due to the anti-fibrotic role of this drug.

## Conclusions

Therefore, in conclusion, our results showed, for the first time, that a pharmacological inhibition of IL-1β could inhibit the development of EMT in renal and hepatic cells and likely inhibits the genesis of chronic organ damage. Although burdened by the limits of an in vitro model, our study emphasizes the therapeutic potential of Canakinumab and further in vivo analyses will be necessary to better characterize this effect. In fact, in the future it is plausible that the pharmacological inhibition of this enzyme may represent a valid therapeutic tool to minimize renal and hepatic fibrosis, especially in transplanted organs from “extended criteria donors” or subjected to multiple pro-fibrotic stimuli.

## Additional file

**Additional file 1: Figure S1.** Gene expression of Metalloproteinase-9 (Mmp-9) in HK-2. **Figure S2.** Enzymatic activity of MMP-9 in HK-2. **Figure S3.** Gene expression of Metalloproteinase-9 (Mmp-9) in LX-2. **Figure S4.** Enzymatic activity of MMP-9 in LX-2.
