# Supplementary material for: In vitro effects of interleukin (IL)-1 beta inhibition on the epithelial-to-mesenchymal transition (EMT) of renal tubular and hepatic stellate cells
Source: J Transl Med. 2019 Jan 7;17:12. doi: 10.1186/s12967-019-1770-1 (PMC6323803; doi:10.1186/s12967-019-1770-1)
Supplement: Supplementary file 1 — Additional file 1: Figure S1. Gene expression of Metalloproteinase-9 (Mmp-9) in HK-2. Figure S2. Enzymatic activity of MMP-9 in HK-2. Figure S3. Gene expression of Metalloproteinase-9 (Mmp-9) in LX-2. Figure S4. Enzymatic activity of MMP-9 in LX-2. [file 12967_2019_1770_MOESM1_ESM.pdf]

Additional file 1

IN VITRO EFFECTS OF INTERLEUKIN (IL)-1 BETA INHIBITION ON THE EPITHELIAL-TO-MESENCHYMAL TRANSITION (EMT) OF RENAL TUBULAR AND HEPATIC STELLATE CELLS.

Valentina Masola*[1], Amedeo Carraro*[2], Simona Granata[1], Lorenzo Signorini[1], Gloria Bellin[1], Paola Violi[2], Antonio Lupo[1], Umberto Tedeschi[2], Maurizio Onisto[3], Giovanni Gambaro[4], Gianluigi Zaza[1].

[1] Renal Unit, Department of Medicine, University-Hospital of Verona, Piazzale Stefani 1, 37126, Verona, Italy;
[2] Department of General Surgery and Odontoiatrics, Liver Transplant Unit, University Hospital of Verona, Piazzale Stefani 1, 37126, Verona, Italy;
[3] University of Padova, Department of Biomedical Sciences, Via Ugo Bassi, 58/B, 35131, Padova, Italy;
[4] Division of Nephrology and Dialysis, Columbus-Gemelli Hospital Catholic University, School of Medicine, Largo Agostino Gemelli 8, 00168, Rome, RM, Italy.

*These authors contributed equally to this work.

Additional Method

Gene expression analysis for Mmp-9

Both cell types were treated with TGF-β (10 ng/ml) or IL-1β (10 ng/ml) in the presence or absence of Canakinumab (5 μg/ml) for 6 hours. Then, whole RNA was extracted using Trizol reagent (Invitrogen), following the manufacturer's instructions. Quantity and quality of RNA were checked using the Nanodrop spectrophotometer (EuroClone). Total RNA was reverse-transcribed into cDNA using the reverse transcriptase SuperScript II (Invitrogen). Real Time-PCR reactions were performed with the ABI-Prism 7500 using Power SYBR Green Master Mix 2 (Applied Biosystem) and specific primers for *Mmp-9* and *Glyceraldehyde-3-phosphate dehydrogenase* (*Gapdh*). The primer sequences for Mmp-9 was: Forward 5'-CCTGGAGACCTGAGAACCAATC-3' and reverse 5'- CCACCCGAGTGTAACCATAGC-3'. The comparative Ct method (ΔΔCt) was used to quantify gene expression, and the relative quantification was calculated as $2^{-\Delta\Delta Ct}$. The GAPDH gene amplification was used as a

reference standard to normalize the target signal. Amplification specificity was controlled by melting curve analysis.

Additional results

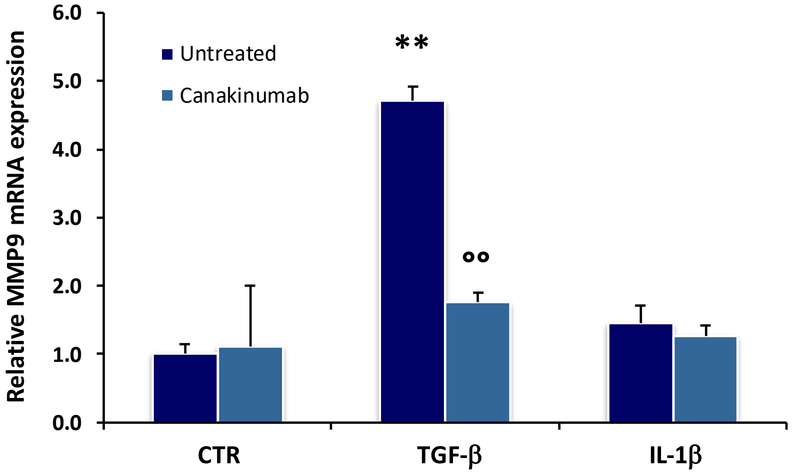

Figure S1. Gene expression of Metalloproteinase-9 (Mmp-9) in HK-2. Gene expression of *Mmp-9* measured by Real Time-PCR in HK-2 cells treated with IL-1β or TGF-β with and without Canakinumab. Expression levels are normalized to *Gapdh*. Data are indicated as mean±SD of three experiments performed in triplicate. The p value was calculated with the t-test. **$p<0.001$ versus untreated control cells (CTR); °°$p<0.01$ vs TGF-β untreated.

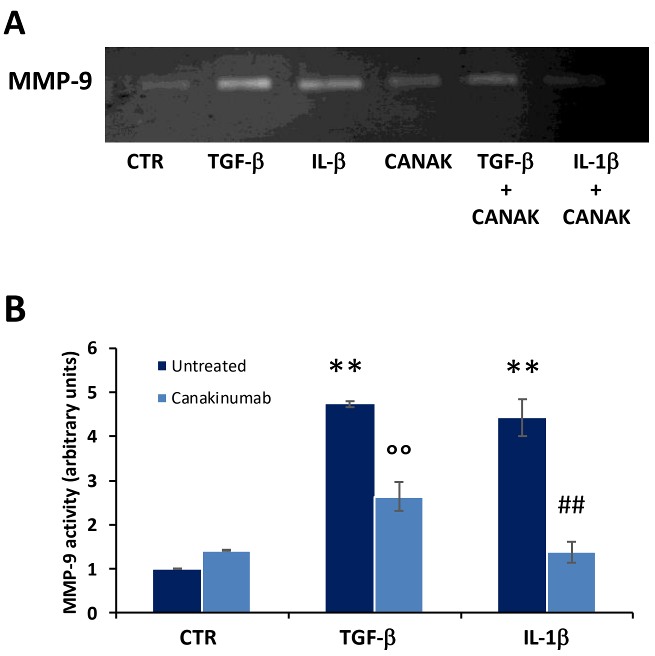

Figure S2. Enzymatic activity of MMP-9 in HK-2. (A) Gelatin zymography shows the activity of MMP-9 in the conditioned media of HK-2 cells treated for 24 h with TGF-β (10 ng/ml) or IL-1β (10 ng/ml) in the presence or absence of Canakinumab (5 μg/ml). **(B)** The histogram represents the densitometric analysis of enzymatic activity of MMP-9 as a mean±SD of three experiments. The p value was calculated with the t-test. **p<0.001 versus untreated control cells (CTR); °°p<0.01 vs TGF-β untreated; ##p<0.01 vs IL-1β untreated.

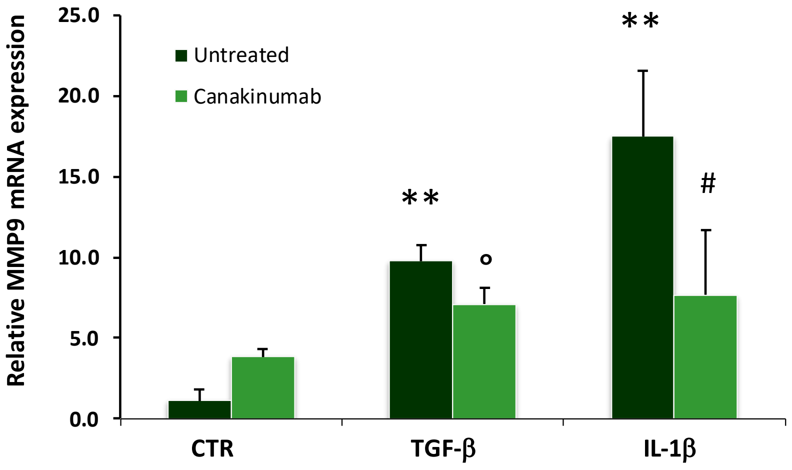

Figure S3. **Gene expression of Metalloproteinase-9 (MMP-9) in LX-2.** Gene expression of *Mmp-9* measured by Real Time-PCR in LX-2 cells treated with IL-1β or TGF-β with and without Canakinumab. Expression levels are normalized to *Gapdh*. Data are indicated as mean±SD of three experiments performed in triplicate. The p value was calculated with the t-test. **p<0.001 versus untreated control cells (CTR); °p<0.05 vs TGF-β untreated; #p<0.05 vs IL-1β untreated.

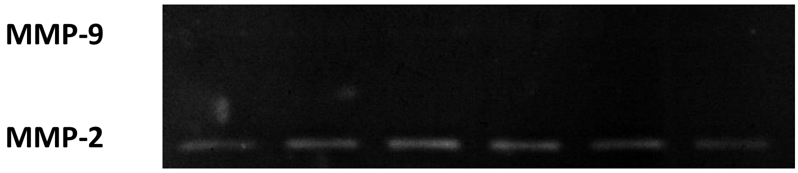

Figure S4. Enzymatic activity of MMP-9 in LX-2. Gelatin zymography shows the activity of MMP-9 in the conditioned media of LX-2 cells treated for 24 h with TGF-β (10 ng/ml) or IL-1β (10 ng/ml) in the presence or absence of Canakinumab (5 μg/ml). MMP-9 activity was not detectable in this cell line.
